# Metabolic Culture Medium Enhances Maturation of Human iPSC-Derived Cardiomyocytes via Cardiac Troponin I Isoform Induction

**DOI:** 10.3390/ijms26157248

**Published:** 2025-07-26

**Authors:** Daria V. Goliusova, Agnessa P. Bogomolova, Alina V. Davidenko, Kristina A. Lavrenteva, Margarita Y. Sharikova, Elena A. Zerkalenkova, Ekaterina M. Vassina, Alexandra N. Bogomazova, Maria A. Lagarkova, Ivan A. Katrukha, Olga S. Lebedeva

**Affiliations:** 1 Lopukhin Federal Research and Clinical Center of Physical-Chemical Medicine, 119435 Moscow, Russia; daria.goliusova@mail.ru (D.V.G.);; 2 Koltzov Institute of Developmental Biology of Russian Academy of Sciences, 119334 Moscow, Russia; 3 Hytest Ltd., 20520 Turku, Finland; 4 Dmitry Rogachev National Medical Research Center of Pediatric Hematology, Oncology and Immunology, 117198 Moscow, Russia; 5 Department of Biochemistry and Molecular Biology, Faculty of General Medicine, Pirogov Russian National Research Medical University, 117997 Moscow, Russia; 6 Department of Biochemistry, Biological Faculty, Lomonosov Moscow State University, 119991 Moscow, Russia

**Keywords:** human iPSCs, iPSC-derived cardiomyocytes, cardiomyocyte maturation, troponin I isoforms, cardiac troponin I, slow skeletal troponin I, cardiac troponin T

## Abstract

Human induced pluripotent stem cell-derived cardiomyocytes (iCMs) provide a powerful platform for investigating cardiac biology. However, structural, metabolic, and electrophysiological immaturity of iCMs limits their capacity to model adult cardiomyocytes. Currently, no universally accepted criteria or protocols for effective iCMs maturation exist. This study aimed to identify practical culture conditions that promote iCMs maturation, thereby generating more physiologically relevant in vitro cardiac models. We evaluated the effects of short- and long-term culture in media supplemented with various stimulatory compounds under 2D conditions, focusing on intracellular content and localization of slow skeletal troponin I (ssTnI) and cardiac troponin I (cTnI) isoforms. Our findings demonstrate that the multicomponent metabolic maturation medium (MM-1) effectively enhances the transition toward a more mature iCM phenotype, as evidenced by increased cTnI expression and formation of cross-striated myofibrils. iCMs cultured in MM-1 more closely resemble adult cardiomyocytes and are compatible with high-resolution single-cell techniques such as electron microscopy and patch-clamp electrophysiology. This work provides a practical and scalable approach for advancing the maturation of iPSC-derived cardiac models, with applications in disease modeling and drug screening.

## 1. Introduction

Since their discovery in 2006 [1], human induced pluripotent stem cells (iPSCs) have become one of the most widely used platforms in both fundamental and translational research [2,3]. A major limitation, however, lies in the immaturity of iPSC-derived cell types compared to their somatic counterparts in vivo. In the absence of external stimuli, iPSC derivatives—including chondrocytes [4], neurons [5], fibroblasts [6], pancreatic β-cells [7], and cardiomyocytes [8]—retain immature characteristics that limit their utility for disease modeling and complicate mechanistic studies in vitro. Over the past decade, extensive efforts have been made to develop effective protocols for the maturation of human iPSC-derived cardiomyocytes (iCMs). Cardiovascular diseases still represent the foremost cause of mortality, contributing to approximately one-third of all deaths worldwide [9]. As the global burden of cardiovascular disease continues to rise—with projections indicating a 90% increase in prevalence between 2025 and 2050 [10]—iCMs have emerged as a valuable model system for cardiac research and development. These cells not only facilitate the study of fundamental heart biology [11] but also offer a promising human-relevant alternative to animal models, particularly for investigating genetic cardiomyopathies [12].

Cardiomyocyte maturation in vivo is a prolonged, multistage process beginning in the perinatal period and continuing into early childhood, encompassing morphological, metabolic, and electrophysiological changes that culminate by 6–10 years of age [13,14,15]. During this process, cardiomyocytes transition from small, round cells into elongated, rod-shaped cells with highly organized myofibrils, widened Z-discs, and developed T-tubules and sarcoplasmic reticulum [16]. Around birth and throughout the neonatal period, cytokinesis becomes decoupled from karyokinesis, causing cells to arrest in the G1 phase of the cell cycle, resulting in binucleation and/or polyploidy. This shift enables hypertrophic heart growth not through proliferation, but through cellular enlargement and ultimately fusion of neighbouring cells, resulting in syncytium formation [17,18,19]. Simultaneously, the metabolic profile switches from glycolysis to fatty acid β-oxidation, providing a more efficient ATP source for postnatal physiology [20,21]. By approximately six years of age, cardiomyocytes exhibit distinct structural localization of gap junction proteins, ensuring continuous ion and molecular transport across intercalated discs and enabling synchronized electrical conduction [13].

Despite advances in differentiation protocols, iCMs in vitro retain a fetal-like phenotype characterized by small, round or irregular morphology, mononuclearity, absence of a branched T-tubule network, and failure to form syncytia. They remain proliferative, rely predominantly on glycolysis, exhibit gene expression profiles typical of fetal cardiomyocytes, and display immature electrophysiological properties, including low resting membrane potential and reduced action potential amplitude [15,22,23,24,25]. The first major challenge in iCM’s maturation lies in the limited understanding of the genetic and regulatory mechanisms governing perinatal cardiomyocyte development. Current maturation strategies aim to recapitulate key structural, metabolic, and electrophysiological transitions by applying physical and chemical stimuli. These include mechanical and/or electrical stimulation, modification of substrate stiffness and geometry, biochemical cues, long-term culture, co-culture, organoid formation, tissue engineering constructs, and in vivo implantation in animal models [8,11,26]. The progress in bioelectrode materials design and microfabrication provides a novel platform for the development of heart-on-a-chip technologies with controlled microenvironments for iCM culture and maturation in vitro [27,28]. In some promising cases, well-designed and implemented maturation strategies have enabled the identification of phenotype-genotype correlations in personalized iCM-based models of inherited cardiomyopathies [29,30,31,32,33,34,35]. However, such successes remain rare, largely due to the high costs and the need for specialized equipment. The second critical bottleneck is the lack of standardized, consensus-based markers for defining iCMs maturity, making it difficult to compare maturation strategies across studies [36,37,38,39]. While comprehensive multiparametric analyses provide the most detailed insights into iCMs maturation, they require interdisciplinary expertise and substantial resources.

This study aimed to identify accessible and effective culture conditions for enhancing the maturation of human iPSC-derived cardiomyocytes, thereby creating a more physiologically relevant in vitro model of myocardium. We focused on accessible approaches to promote iCMs maturation, adopting a widely used strategy involving culture in media supplemented with maturation-promoting factors. As a first-tier maturation marker, we selected the ratio of slow skeletal troponin I (ssTnI) to cardiac troponin I (cTnI)—a robust, in vivo-validated indicator proposed by Bedada et al. in 2014—as a quantifiable measure of structural iCMs maturation across laboratories [14]. We supplemented this with analysis of the intracellular localization of cTnI and ssTnI at the single-cell level to further assess structural maturation.

## 2. Results

### 2.1. Reprogramming of Dermal Fibroblasts to a Pluripotent State

**Figure 1:**
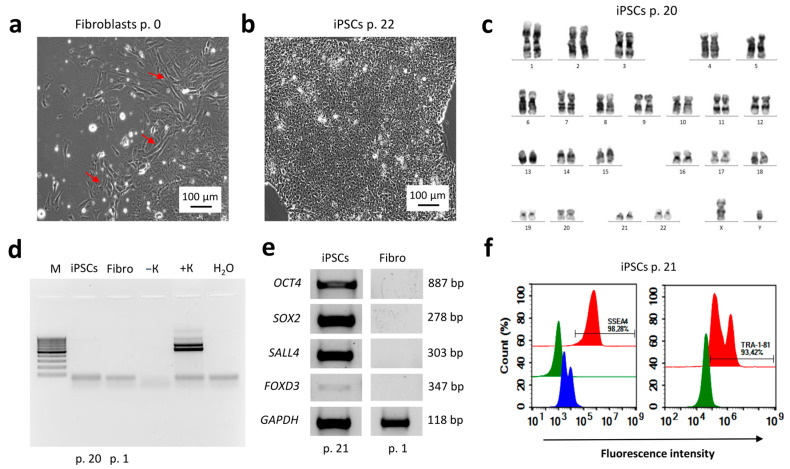
Molecular and genetic characterization of the RCPCMi013-A (IPSAVE2S) iPSC line. (**a**) Morphology of primary dermal fibroblasts isolated from a skin biopsy (indicated by red arrows); (**b**) morphology of an iPSC colony; (**c**) karyogram of iPSCs; (**d**) electropherogram of amplification products for the *Mycoplasma* spp. 16S rRNA gene in iPSCs (M—DNA length marker, Fibro—original dermal fibroblasts, −K—negative control, +K—positive control); (**e**) electrophoregram of amplification products for pluripotency genes *OCT4*, *SOX2*, *SALL4*, *FOXD3*, and the housekeeping gene *GAPDH* in iPSCs and the original fibroblasts; (**f**) flow cytometry data showing expression of surface pluripotency markers SSEA4 (left) and TRA-1-81 (right) in iPSCs (unstained cells are shown in green, isotype control in blue, stained cells in red). p.—cell passage; bp—base pairs.

A primary dermal fibroblast culture (Figure 1a) was established from a skin biopsy of a healthy donor. A total of 1.5 × 10^5^ passage 1 dermal fibroblasts were transduced with the four Yamanaka reprogramming factors. Approximately three weeks after transduction, colonies exhibiting the typical morphology of human pluripotent stem cells (hPSCs) began to emerge. In total, 12 colonies were selected. The reprogramming efficiency, calculated as the ratio of colonies formed to the number of transduced cells, was 0.008%. Molecular and functional characterization of the resulting iPSC line, RCPCMi013-A (IPSAVE2S), confirmed its pluripotent status. The cells exhibited typical hPSC morphology (Figure 1b), a normal 46, XY karyotype (Figure 1c), and genetic identity to the parental fibroblasts across 19 STR markers and the amelogenin locus (data available upon request). They were negative for *Mycoplasma* spp. contamination (Figure 1d) and expressed the core pluripotency genes *OCT4*, *SOX2*, *SALL4*, and *FOXD3* (Figure 1e), as well as the surface markers SSEA4 and TRA-1-81 (Figure 1f). The resulting RCPCMi013-A (IPSAVE2S) cell line passport is provided in Supplementary Table S1.

**Figure 2:**
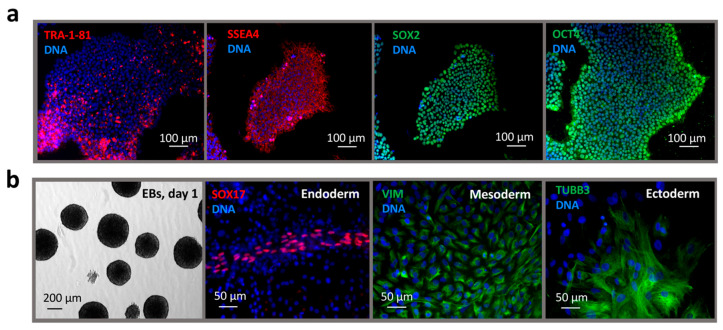
Molecular and functional characterization of the RCPCMi013-A (IPSAVE2S) iPSC line. (**a**) Immunostaining for nuclear (OCT4, SOX2) and surface (SSEA4, TRA-1-81) pluripotency markers; (**b**) morphology of embryoid bodies (EBs) derived from iPSCs and immunostaining of EBs-derived cells for markers of the three germ layers: SOX17 (endoderm), VIM (mesoderm), and TUBB3 (ectoderm). Cell nuclei are counterstained with DAPI.

Immunostaining confirmed expression of nuclear (OCT4, SOX2) and surface (SSEA4, TRA-1-81) pluripotency markers (Figure 2a). The IPSAVE2S cells were capable of forming embryoid bodies and differentiating spontaneously into derivatives of all three germ layers. Immunostaining showed positive expression of SOX17 (endoderm), vimentin (VIM, mesoderm), and β-III-tubulin (TUBB3, ectoderm) (Figure 2b).

### 2.2. Differentiation of iPSCs into Cardiomyocytes

**Figure 3:**
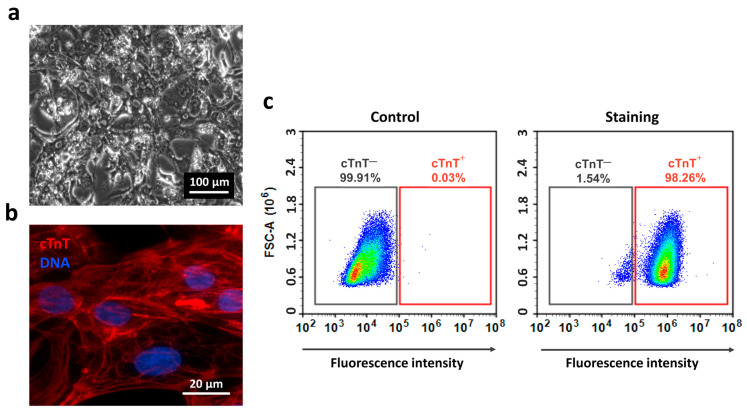
AVE2S-iCMs morphology and cardiac troponin T expression. (**a**) Cell morphology under phase contrast (passage 1, day 22); (**b**) immunocytochemical staining for cTnT (passage 2, day 30); cell nuclei are counterstained with DAPI; (**c**) flow cytometry fluorescence density plots illustrating the proportion of cTnT-negative cells (cTnT^—^, grey rectangle) and cTnT-positive cells (cTnT^+^, red rectangle) in iCMs culture (passage 1, day 22). FSC-A—forward scatter area.

To assess the cardiogenic differentiation potential of the IPSAVE2S line, we conducted a pilot differentiation experiment aimed at generating and characterizing cardiomyocytes. For four passages prior to the start of differentiation, IPSAVE2S cells were passaged at a consistent density of 0.35 × 10^5^ cells/cm^2^ every three days. At passage 18, the cells were seeded at a density of 1.25 × 10^5^ cells/cm^2^ and subjected to differentiation using the commercial STEMdiff Ventricular Cardiomyocyte Differentiation Kit according to the manufacturer’s protocol. By day 8, the first clusters of spontaneously contracting iCMs were observed. Between days 10 and 14, non-myocyte populations present in the culture were selectively eliminated through metabolic stress selection in a glucose-free medium supplemented with lactate. This selection step led to the death of cells unable to utilize lactate as a primary energy source, while the iCMs monolayer exhibited spontaneous contractile activity (Supplementary Video S1). Subsequently, the cells were cultured in STEMdiff Cardiomyocyte Maintenance Medium. By day 22, cultures consisted predominantly of spontaneously contracting iCMs displaying light nuclear and dark cytoplasmic morphology (Figure 3a). Immunostaining revealed strong sarcomeric cross-striation for cardiac troponin T (cTnT) (Figure 3b), and flow cytometry indicated 98% positivity compared to isotype controls (Figure 3c). These results demonstrate that the IPSAVE2S line differentiates efficiently into cardiomyocytes.

### 2.3. Maturation and Comparative Analysis of iCMs Maturity

**Figure 4:**
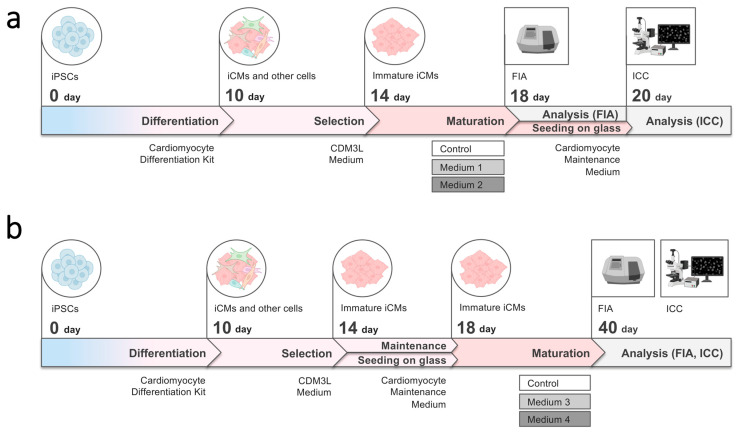
Schematic representation of human iCMs derivation and maturation protocols. (**a**) Short-term (4-day) maturation experiment; (**b**) long-term (22-day) maturation experiment. FIA—fluoroimmunoassay; ICC—immunocytochemical staining.

To analyze cTnI, ssTnI, and cTnT quantitative content, cTnI/ssTnI ratios, and intracellular localization pattern of cTnI and ssTnI in human iCMs, we differentiated wild-type hiPSCs (IPSAVE2S) using the STEMdiff Ventricular Cardiomyocyte Differentiation Kit and performed a short-term (4-day) or long-term (22-day) maturation of the resulting iCM cultures following metabolic selection (Figure 4). We used four types of maturation media containing maturation-promoting factors (Table 1). The unmodified commercial STEMdiff Cardiomyocyte Maintenance Medium was used as the control condition in all experiments.

In the first experiment, AVE2S-iCMs were derived from passage 31 of IPSAVE2S. Short-term maturation was performed over 4 days using the STEMdiff Cardiomyocyte Maintenance Medium supplemented either with T3, IGF-1, dexamethasone, and lipid concentrate (Medium 1) or with etoposide and lipid concentrate (Medium 2) (Figure 4a). In the second experiment, AVE2S-iCMs were derived from passage 38 of IPSAVE2S. Maturation was carried out over 22 days in either RPMI-1640 with standard glucose levels supplemented with T3, IGF-1, dexamethasone, and NeuroMax (Medium 3), or in low-glucose DMEM supplemented with L-glutamine, non-essential amino acids, sodium lactate, vitamin B12, biotin, creatine, L-taurine, L-carnitine, ascorbic acid, human albumin, lipid concentrate, NeuroMax, and KnockOut SR (Medium 4) (Figure 4b). At the end of the experiments, iCMs were fixed for immunostaining and collected for fluoroimmunoassay. Quantitative data on cell counts, concentrations of cTnI, ssTnI, and cTnT, as well as cTnI/ssTnI ratios in the resulting AVE2S-iCMs samples are summarized in Supplementary Table S2.

**Figure 5:**
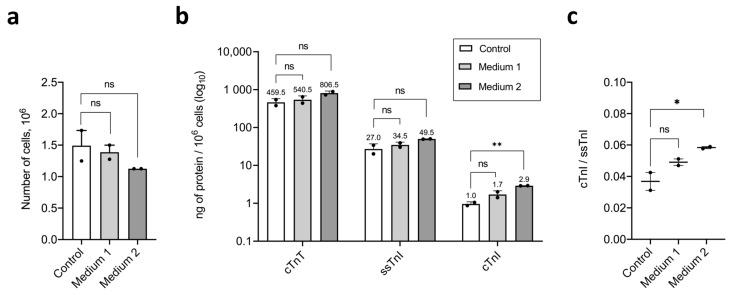
Troponin content in human iCMs after short-term maturation in Media 1 and 2. (**a**) Number of live cells in samples collected for fluoroimmunoassay; (**b**) content of troponins (cTnT, ssTnI, and cTnI) per 10^6^ cells in the analyzed samples; (**c**) ratio of TnI isoforms (cTnI/ssTnI), expressed in ng of protein per 10^6^ cells. Data are presented as mean ± SD. ns—not significant; * *p* < 0.05; ** *p* < 0.005.

Compared with control conditions, culture in Medium 1 did not result in statistically significant differences in live cell count (1.4915 × 10^6^ cells in control vs. 1.3875 × 10^6^ cells in Medium 1) (Figure 5a), cTnT content (459.825 ng/10^6^ cells in control vs. 540.645 ng/10^6^ cells in Medium 1), ssTnI content (27.065 ng/10^6^ cells in control vs. 34.64 ng/10^6^ cells in Medium 1), or cTnI content (0.96 ng/10^6^ cells in control vs. 1.71 ng/10^6^cells in Medium 1) (Figure 5b) in the iCMs cultures at the end of the experiment. After maturation in Medium 1, the ssTnI level remained 20 times higher than that of cTnI (34.64 ng/10^6^ cells for ssTnI vs. 1.71 ng/10^6^ cells for cTnI), while in the control, this difference was 28.2-fold (27.065 ng/10^6^ cells for ssTnI vs. 0.96 ng/10^6^ cells for cTnI) (Figure 5b). The cTnI/ssTnI ratio remained <1:0.049 in Medium 1 and 0.037 in control, with no significant difference (Figure 5c).

Similarly, Medium 2 did not affect live cell count (1.4915 × 10^6^ cells in control vs. 1.125 × 10^6^ cells in Medium 2) (Figure 5a), cTnT content (459.825 ng/10^6^ cells in control vs. 806.575 ng/10^6^ cells in Medium 2), or ssTnI content (27.065 ng/10^6^ cells in control vs. 49.745 ng/10^6^ cells in Medium 2), but did result in a 3-fold increase in cTnI content (0.96 ng/10^6^ cells in control vs. 2.9 ng/10^6^ cells in Medium 2) (Figure 5b). After maturation in Medium 2, the ssTnI level remained 17 times higher than that of cTnI (49.745 ng/10^6^ cells for ssTnI vs. 2.9 ng/10^6^ cells for cTnI), while in the control, this difference was 28.2-fold (27.065 ng/10^6^ cells for ssTnI vs. 0.96 ng/10^6^ cells for cTnI) (Figure 5b). The cTnI/ssTnI ratio remained below 1 (0.059), however, increased slightly versus control (0.037) (Figure 5c).

**Figure 6:**
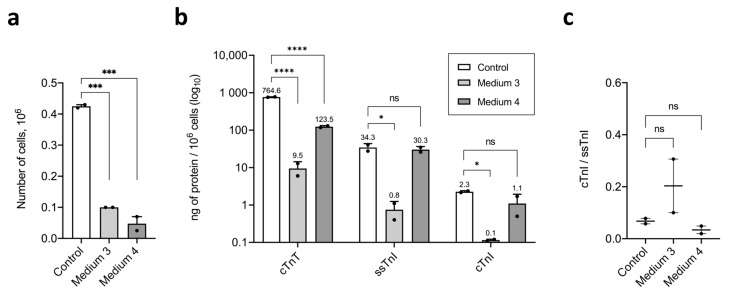
Troponin content in human iCMs after long-term maturation in Media 3 and 4. (**a**) Number of live cells in samples collected for fluoroimmunoassay; (**b**) content of troponins (cTnT, ssTnI, cTnI) per 10^6^ cells in the analyzed samples; (**c**) ratio of TnI isoforms (cTnI/ssTnI), expressed in ng of protein per 10^6^ cells. Data are presented as mean ± SD. ns—not significant; * *p* < 0.05; *** *p* < 0.0005; **** *p* < 0.0001.

Compared with the control, culture in Medium 3 led to a 4.25-fold decrease in cell number (0.425 × 10^6^ cells in control vs. 0.1 × 10^6^ cells in Medium 3) (Figure 6a), 80.9-fold reduction in cTnT content (764.59 ng/10^6^ cells in control vs. 9.45 ng/10^6^ cells in Medium 3), 44.8-fold reduction in ssTnI content (34.305 ng/10^6^ cells in control vs. 0.765 ng/10^6^ cells in Medium 3), and 19.7-fold reduction in cTnI content (2.26 ng/10^6^ cells in control vs. 0.115 ng/10^6^ cells in Medium 3) (Figure 6b). Following maturation, ssTnI levels remained 6.7 times higher than cTnI (0.765 ng/10^6^ cells for ssTnI vs. 0.115 ng/10^6^ cells for cTnI), while in the control, this ratio was 15.2-fold (34.305 ng/10^6^ cells for ssTnI vs. 2.26 ng/10^6^ cells for cTnI) (Figure 6b). The cTnI/ssTnI ratio was 0.203, not significantly different from control (0.068) (Figure 6c).

The culture in Medium 4 (MM-1) resulted in an 8.9-fold reduction in cell count (0.425 × 10^6^ cells in control vs. 0.0475 × 10^6^ cells in Medium 4) (Figure 6a) and a 6-fold reduction in cTnT content (764.59 ng/10^6^ cells in control vs. 123.42 ng/10^6^ cells in Medium 4), while ssTnI (34.305 ng/10^6^ cells in control vs. 30.315 ng/10^6^ cells in Medium 4) and cTnI (2.26 ng/10^6^ cells in control vs. 1.095 ng/10^6^ cells in Medium 4) levels were not significantly changed (Figure 6b). After maturation in MM-1, ssTnI remained 27.7 times higher than cTnI (30.315 ng/10^6^ cells for ssTnI vs. 1.095 ng/10^6^ cells for cTnI), while in the control, this ratio was 15.2-fold (34.305 ng/10^6^ cells for ssTnI vs. 2.26 ng/10^6^ cells for cTnI) (Figure 6b). The cTnI/ssTnI ratio was 0.034, again not significantly different from the control 0.068 value (Figure 6c).

**Figure 7:**
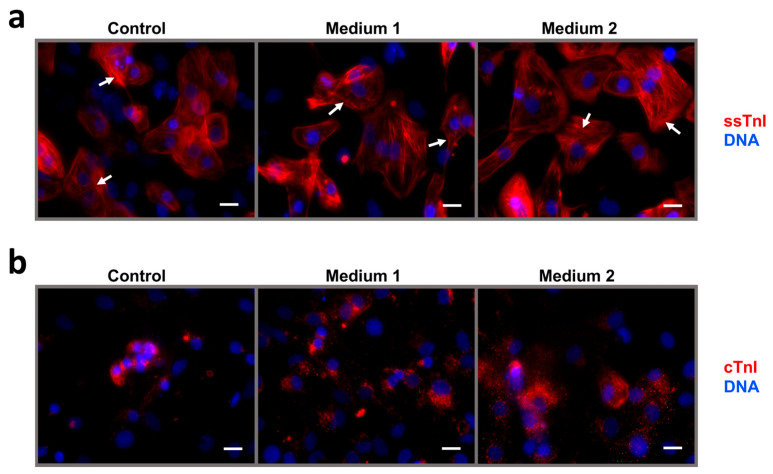
Intracellular localization of troponin I isoforms in human iCMs after short-term maturation in Media 1 and 2. (**a**) ssTnI; (**b**) cTnI. Cell nuclei are counterstained with DAPI. White arrows indicate iCMs myofibrils exhibiting a cross-striated pattern. Scale bar: 20 µm.

**Figure 8:**
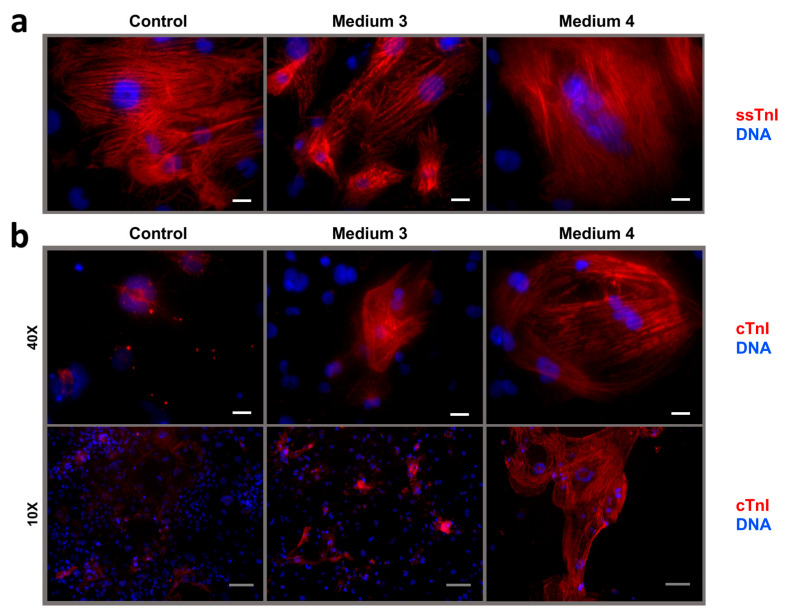
Intracellular localization of troponin I isoforms in human iCMs after long-term maturation in Media 3 and 4 (MM-1). (**a**) ssTnI; (**b**) cTnI. Cell nuclei are counterstained with DAPI. White scale bar: 20 µm; grey scale bar: 100 µm. Magnification: 40× and 10× objectives.

Immunostaining for ssTnI revealed well-organized sarcomeric striations in all iCMs in the short-term maturation experiment (Figure 7a). In contrast, cTnI staining remained diffuse across all samples, though signal intensity trended upward from control to Medium 2 (Figure 7b). In the long-term maturation experiment, immunostaining for ssTnI revealed the same well-organized sarcomeric striations in all iCMs (Figure 8a). Unexpectedly, immunostaining for cTnI revealed isolated cTnI^+^-iCMs with cross-striation following maturation in Medium 3, and clusters of large cTnI^+^-iCMs with clear striation in Medium MM-1 (Figure 8b).

Thus, in the short-term maturation experiment, no significant differences in the number of live cells collected for fluoroimmune analysis were observed across all iCM cultures. Levels of cTnT, ssTnI, and cTnI were similar between treated and control cultures, except for a modest increase in cTnI in cells exposed to etoposide and lipid concentrate. The cTnI/ssTnI ratio remained below 1 in all conditions: 0.037 (control), 0.049 (Medium 1), and 0.059 (Medium 2). Treatment with Medium 2 resulted in a slight but statistically significant increase in the cTnI/ssTnI ratio. Immunostaining for ssTnI revealed a cross-striated pattern of myofibrils in all iCM cultures. In contrast, cTnI staining showed diffuse cytoplasmic localization, with a trend toward signal enhancement from control to Medium 1 to Medium 2, but without detectable cross-striation as a marker of iCM’s structural maturity.

In summary, the long-term maturation experiment revealed a significant reduction in live cell count in both matured iCM cultures compared to the control. In Medium 3, all troponins—cTnT, ssTnI, and cTnI—were significantly reduced, whereas in MM-1, only the cTnT level decreased. The cTnI/ssTnI ratio remained below 1 in all conditions: 0.068 (control), 0.203 (Medium 3), and 0.034 (MM-1). Immunostaining for ssTnI demonstrated a cross-striated pattern of iCM myofibrils in the analyzed cell cultures. Unexpectedly, we observed isolated mature cTnI^+^-iCMs with evident cross-striations of sarcomeres in Media 3 and clustered mature cTnI^+^-iCMs with evident cross-striations of sarcomeres in MM-1.

## 3. Discussion

Globally, considerable effort is being focused on developing strategies for the maturation of cardiomyocytes derived from human induced pluripotent stem cells (iCMs), aiming to improve the physiological relevance of cardiac models. Despite progress in identifying transcriptional regulators involved in cardiomyocyte maturation both in vivo [25] and in vitro [40,41], our understanding of the key upstream triggers and master regulators remains incomplete. Consequently, no universal strategy currently exists to activate the cardiomyocyte maturation program and reproducibly induce the formation of structurally and functionally mature iCMs in vitro. Most current maturation protocols focus not on gene expression modulation, but on biophysical or engineering interventions—such as 3D tissue constructs, mechanical or electrical stimulation—which often require specialized equipment and interdisciplinary resources. Among more accessible approaches, supplementation of 2D cultures with hormonal, metabolic, or signaling factors remains one of the most practical strategies [11,26].

In this study, we systematically evaluated four media formulations designed to promote cardiomyocyte maturation in 2D culture by short-term or long-term exposure to stimulatory compounds. Our objective was to identify a robust and scalable approach that can be integrated into standard protocols for producing patient-specific or healthy iCMs suitable for rare cardiomyopathy modeling. As a foundation, we derived and characterized a new wild-type iPSC line (IPSAVE2S) from dermal fibroblasts using a non-integrating Sendai virus system delivering hOCT3/4, hSOX2, hKLF4, and hc-MYC [42], which is known for its high reprogramming efficiency and low incidence of chromosomal abnormalities [43]. The iPSC line met established pluripotency criteria [44].

For differentiation, we selected a reproducible protocol optimized for ventricular cardiomyocyte generation with a stated efficiency > 80%, based on cTnT expression. To enhance culture homogeneity, we incorporated a 4-day metabolic selection phase exploiting glucose deprivation, which selectively eliminates non-cardiomyocyte lineages [45]. This approach yielded a highly pure cTnT^+^-iCMs population.

As a first-line marker of structural maturation, we employed the ratio of cTnI to ssTnI, a well-established developmental metric. In mammalian heart development, including humans, only ssTnI is expressed during early stages. Expression of cTnI begins between 20 and 33 weeks of gestation, with an approximate 1:1 ratio at birth. A permanent isoform switch favoring cTnI occurs postnatally, and in the adult heart, only cTnI is expressed [46,47,48,49]. Thus, the cTnI/ssTnI ratio serves as a quantitative indicator of iCMs maturity: <1 in fetal-like iCMs, ~1 in neonatal, and >1 in mature postnatal cardiomyocytes [14]. Unlike many other markers, this ratio can be measured at both the population and single-cell levels across all stages of culture.

As an internal control, we also measured cTnT, a cardiomyocyte-specific component of the sarcomeric troponin complex [50]. cTnT is the only troponin T isoform expressed in human cardiomyocytes throughout ontogeny [51]. Since we used antibodies targeting constitutively expressed cTnT epitopes [52], we did not distinguish between embryonic and adult splice isoforms of cTnT [53,54]. All troponin levels were quantified using a highly sensitive and specific sandwich fluoroimmunoassay [55,56,57].

Among stimulatory maturation factors, we used fatty acids, T3, IGF-1, dexamethasone, etoposide, and multicomponent metabolic maturation medium 1 (MM-1). Medium 1 (T3, IGF-1, dexamethasone, and fatty acids) and Medium 2 (etoposide and fatty acids) were prepared using the commercial STEMdiff Cardiomyocyte Maintenance Medium as the basal formulation. Medium 3 (T3, IGF-1, dexamethasone, and NeuroMax) was based on standard-glucose RPMI-1640 [33]. The 12-component Medium 4 (MM-1) was based on glucose-free DMEM, following the published metabolic maturation medium protocol [31].

### 3.1. Fatty Acids

Fatty acids are the primary energy source for adult human cardiomyocytes [20,21]. In contrast, standard culture media typically rely on glucose (10–25 mM) as the dominant metabolic substrate and contain only minimal lipid content. For example, RPMI medium supplemented with B27 and insulin—a commonly used formulation for iCM culture—contains less than 10 μM total lipids [58], whereas the concentration of circulating fatty acids in neonatal human serum is approximately 300 μM [59]. To mimic the metabolic environment of the postnatal heart and promote oxidative metabolism, one established strategy for iCMs maturation involves supplementing culture media with exogenous fatty acids—such as palmitate, oleate, or linoleic acid—and reducing glucose levels or replacing glucose with galactose to suppress glycolysis [33,60]. In this study, we applied a chemically defined lipid concentrate containing both saturated and unsaturated fatty acids, in combination with additional stimulatory compounds, to support metabolic maturation of iCMs.

### 3.2. T3, IGF-1, and Dexamethasone

Culturing iCMs in media supplemented with hormones and fatty acids is a widely used strategy that mimics key aspects of in vivo cardiac development [61,62,63]. The thyroid hormone T3 and the glucocorticoid dexamethasone are critical for neonatal adaptation and exhibit a surge in both concentration and biological activity during late gestation [64,65,66]. IGF-1 is also a key developmental regulator of cardiomyocyte growth and structural remodeling [67,68]. Prior studies have shown that combined treatment with T3, IGF-1, and dexamethasone synergistically enhances the functional maturation of iCMs [29].

### 3.3. Etoposide

Etoposide is a semisynthetic derivative of podophyllotoxin, a compound extracted from *Podophyllum peltatum* roots, and has not previously been applied to promote iCMs maturation. It acts as a chemotherapeutic agent through inhibition of topoisomerase II, leading to DNA strand breaks and impaired DNA synthesis [69,70]. In a study investigating etoposide-induced cardiotoxicity, Nemade et al. showed that treatment with 10–30 μM etoposide activated transcriptional and miRNA programs associated with mitochondrial remodeling, contractile protein regulation, and myocardial hypertrophy in human iCMs. However, 48 h treatment at concentrations of 10, 15, or 30 μM did not affect expression of *TNNI3*, which encodes cTnI [71]. In this study, we addressed the question of whether a prolonged (96 h) 25 μM etoposide exposure can stimulate TnI isoforms switch and cTnI expression at the protein level in iCMs without significant toxic side-effects.

### 3.4. Metabolic Maturation Medium

In 2020, Feyen et al. introduced a metabolic maturation medium (MM) designed to promote oxidative metabolism and enhance the physiological function of iCMs [31]. This formulation is based on DMEM with reduced glucose (3 mM) and elevated lactate (10 mM), supplemented with vitamin B12, biotin, creatine, taurine, carnitine, ascorbic acid, fatty acids bound to bovine serum albumin (AlbuMAX), B27, KnockOut SR, and essential amino acids. Culturing iCMs in MM for 3–5 weeks was shown to enhance metabolic activity, electrophysiology, and contractile function. Cells exhibited increased expression of genes related to ion channel function and mitochondrial metabolism, including genes involved in cristae formation, alongside enhanced contractile force and increased reliance on Na^+^ and Ca^2+^ currents [31]. More recently, Fetterman et al. validated the effectiveness of similar multicomponent maturation media in promoting various aspects of iCMs development [72].

### 3.5. Comparative Effects of Experimental Conditions

We found that 96 h exposure to 25 μM etoposide (Medium 2) resulted in a detectable increase in cTnI protein expression without significant change in the number of viable cells in culture. However, this increase was insufficient to drive complete isoform switching from slow skeletal TnI to cardiac TnI; the cTnI/ssTnI ratio remained below 1, consistent with a fetal-like phenotype. Given this partial effect, it may be of interest to evaluate whether a more prolonged etoposide exposure could further promote structural maturation of iCMs. Nevertheless, the use of etoposide, e.g., in patient-specific iCMs is questionable, as its cytotoxic effects may distort disease-relevant molecular profiles and compromise cell viability.

Neither short-term (4-day) combined treatment with T3, IGF-1, dexamethasone, and fatty acids (Medium 1), nor prolonged (22-day) exposure to T3, IGF-1, dexamethasone, and NeuroMax (Medium 3) promoted isoform switching from ssTnI to cTnI across the iCMs culture. In both conditions, the cTnI/ssTnI ratio remained below 1, indicating that the cells retained a fetal-like phenotype. Immunocytochemical staining for ssTnI and cTnI suggested early signs of maturation and modest upregulation of cTnI expression in response to Medium 1; however, four days of treatment were insufficient to drive isoform switching. Interestingly, extending the duration of T3, IGF-1, and dexamethasone exposure (Medium 3) enhanced maturation at the single-cell level: for the first time, we detected individual cTnI^+^-iCMs with clear sarcomeric cross-striations. At the same time, however, this prolonged treatment resulted in a marked loss of cell viability; therefore, further use of Medium 3 was discontinued. It may still be valuable to assess this hormonal combination under alternative basal conditions, such as STEMdiff Cardiomyocyte Maintenance Medium, to mitigate cytotoxic effects.

Medium 4 was prepared according to the published formulation for metabolic maturation of iCMs [31], with functional substitution of two components: AlbuMAX (fatty acids bound to bovine serum albumin) and B27 supplement were replaced with a chemically defined lipid concentrate (containing both saturated and unsaturated fatty acids), recombinant human albumin, and the NeuroMax supplement (B27 analogue). Our results show that culturing iCMs in MM-1 for 22 days did not induce global isoform switching from ssTnI to cTnI; the cTnI/ssTnI ratio remained below one across the culture, consistent with a fetal-like phenotype. However, compared to other tested conditions, this approach most effectively promoted cTnI expression at the single-cell level, indicating localized isoform transition. Only in MM-1–treated cultures did we observe clusters of cTnI^+^-iCMs exhibiting well-defined sarcomeric cross-striations.

These findings suggest that MM-1 promotes structural maturation in a subpopulation of cells, even in the absence of a culture-wide shift in TnI isoform expression. Future studies should include a quantitative assessment of cTnI^+^-iCMs in MM-1 cultures under extended treatment durations and/or refined medium composition, such as the addition of galactose, insulin-transferrin-selenium supplement, T3, dexamethasone, or other supportive factors.

## 4. Materials and Methods

### 4.1. Cell Material and Troponin-Specific Antibodies

Biological material was obtained from a healthy donor who provided informed consent prior to study inclusion. Monoclonal antibodies (mAbs) specific to troponins—skTnI58, skTnI27, skTnI30 [55], Y306, Y603, MF4, 329, 406—along with recombinant human cardiac troponin ITC-complex (cITC) and recombinant human slow skeletal troponin IC-complex (ssIC) were kindly provided by HyTest (Turku, Finland).

### 4.2. Isolation and Culture of Fibroblasts

Dermal fibroblasts were isolated from a skin biopsy (~1 cm^2^) of a healthy 45-year-old male donor. The biopsy was cut into 2–3 mm^2^ fragments, placed on a 60-mm Petri dish lid (SPL Life Science, Pocheon, Republic of Korea) in a drop of DMEM (PanEco, Moscow, Russia) containing 20 μg/mL gentamicin (PanEco, Russia). Fragments were transferred onto 35-mm Petri dishes (SPL Life Science, Pocheon, Republic of Korea) and covered with a 24 × 24 mm coverslip (Menzel-Gläser, Braunschweig, Germany). Fibroblast culture medium composed of DMEM (PanEco, Russia), 20% fetal bovine serum (FBS) (HyClone, Hyde Park, UT, USA), 1% MEM non-essential amino acids (PanEco, Russia), 2 mM alanyl-glutamine (PanEco, Russia), 10 μg/mL gentamicin, 50 U/mL penicillin, and 50 μg/mL streptomycin (PanEco, Russia) was added (3 mL per dish). Cultures were maintained at 37 °C, 5% CO_2_. Primary keratinocytes emerged from explants within 3–5 days, followed by fibroblasts. From day 5 onward, the medium was changed every 3 days, reducing FBS concentration to 10%. Upon reaching 100% confluency, fibroblasts were passaged using 0.25% trypsin-EDTA solution (Gibco, New York, NY, USA): wells were washed twice with 1 mL of Hank’s solution (PanEco, Russia), then 1 mL of trypsin was added. Cells were incubated in a CO_2_ incubator for 5–8 min under microscopic control. Cells were then resuspended, and trypsin was inactivated by adding an equal volume of DMEM (PanEco, Russia) supplemented with 10% FBS (HyClone, USA). The cell suspension was centrifuged for 5 min at 200× *g* (Eppendorf Centrifuge 5804R, Eppendorf, Hamburg, Germany). Cell counting was performed using an improved Neubauer chamber C-Chip (INCYTO, Cheonan, Republic of Korea) with 10 µL of a single-cell suspension stained 1:1 with trypan blue (PanEco, Russia). Fibroblasts were seeded at a density of 0.05 × 10^5^ cells/cm^2^ onto six-well culture plates (SPL Life Science, Republic of Korea) or 35-mm Petri dishes (SPL Life Science, Republic of Korea) in fibroblast culture medium. For cryopreservation cell pellets were resuspended in 1 mL of DMEM (PanEco, Russia) containing 10% FBS (HyClone, USA) and 10% DMSO (PanEco, Russia), transferred into cryovials (SPL Life Science, Republic of Korea), cooled at −70 °C for 24 h in a controlled-rate freezer (Sanyo, Osaka, Japan) and subsequently stored in liquid nitrogen.

### 4.3. Generation and Culture of iPSCs

Passage 1 dermal fibroblasts cultured on 35-mm Petri dish (SPL Life Science, Republic of Korea) were reprogrammed into induced pluripotent stem cells (iPSCs) by transduction with pluripotency factors hKOS (vector carrying hKLF4, hOCT3/4, and hSOX2), hc-MYC, and hKLF4 using the CytoTune iPS 2.0 Sendai Reprogramming Kit (ThermoFisher Scientific, Waltham, MA, USA) according to the manufacturer’s protocol. Colonies were manually picked and transferred to Matrigel-coated wells (Matrigel hES-qualified matrix, Corning, Corning, NY, USA) of 24-well culture plates (SPL Life Science, Republic of Korea) in mTeSR1 medium (Stemcell Technologies, Vancouver, BC, Canada) supplemented with 5 μM ROCK inhibitor Y-27632 (Stemcell Technologies, Canada). After 24 h, the culture medium was changed to mTeSR1. iPSC clones were cultured until expansion in mTeSR1 with daily medium change. Upon 70–80% confluence, iPSCs were passaged using 0.05% trypsin-EDTA solution (Gibco, USA). The cell suspension was centrifuged for 5 min at 200× *g* (Eppendorf Centrifuge 5804R). Viable cells were counted using an improved Neubauer chamber C-Chip (INCYTO, Republic of Korea) with 10 µL of single-cell suspension stained 1:1 with trypan blue (PanEco, Russia). iPSCs were plated at a 1:4 split ratio at 0.3–0.5 × 10^5^ cells/cm^2^ on Matrigel-coated culture plastic in culture medium containing a 4:1 mixture of GibriS-8 (PanEco, Russia) and mTeSR1 (GibriS-8/mTeSR1) supplemented with 5 μM ROCK inhibitor Y-27632 (Stemcell Technologies, Canada). After 24 h culture medium was changed to GibriS-8/mTeSR1. iPSCs were cultured in the presence of 50 U/mL penicillin and 50 μg/mL streptomycin (PanEco, Russia) with daily medium change. For cryopreservation, 5–8 × 10^5^ cells were resuspended in 0.5 mL fetal bovine serum (FBS) (Himedia, Mumbai, India), transferred into cryovials, mixed with 0.5 mL FBS containing 20% dimethyl sulfoxide (DMSO) (PanEco, Russia), gently mixed once, and stored at –70 °C for 24 h before transfer to liquid nitrogen for long-term storage.

### 4.4. Validation of iPSC Line

Genetic, molecular, and functional characterization of iPSCs was performed using primary antibodies against TRA-1-81 (Santa Cruz Biotechnology, Dallas, TX, USA, Cat.#sc-21706, 1:15 dilution) (Flow cytometry), Vimentin (Invitrogen, ThermoFisher Scientific, USA, Cat.#MA5-11883, 1:250 dilution), and SOX17 (Abcam, Cambridge, U.K., Cat.#ab224637, 1:100 dilution) (Immunocytochemical staining) as described previously [73].

### 4.5. Differentiation and Culture of iPSC-Derived Cardiomyocytes

iPSCs were differentiated into ventricular iCMs on 12-well culture plates (SPL Life Science, Republic of Korea) under standard conditions (37 °C, 5% CO_2_) in the presence of 50 U/mL and 50 µg/mL penicillin-streptomycin (PanEco, Russia) using the STEMdiff Ventricular Cardiomyocyte Differentiation Kit (Stemcell Technologies, Canada) following the manufacturer’s protocol. At the end of differentiation (days 10–14), metabolic selection of iCMs was performed for 4 days in CDM3L medium composed of glucose- and glutamine-free RPMI-1640 (PanEco, Russia), 0.5 mg/mL recombinant human albumin (eEnzyme, Gaithersburg, MD, USA), 1.2 mM ascorbic acid (Sigma-Aldrich, St. Louis, MO, USA), 4 mM sodium lactate (PanReac, Barcelona, Spain), supplemented with 1× Glutamax (Gibco, USA) or 1.84 mM alanyl-glutamine (PanEco, Russia). iCMs were passaged using 0.25% trypsin-EDTA solution (PanEco, Russia): wells were washed twice with Hank’s solution (PanEco, Russia), trypsin was added and incubated for 6–10 min under microscopic control, cells were resuspended into a single-cell suspension, trypsin was inactivated by twice the volume of DMEM (PanEco, Russia) with 10% FBS (Himedia, India), and centrifuged for 5 min at 200× *g* (Eppendorf Centrifuge 5804R, Eppendorf, Germany). The pellet was resuspended in STEMdiff Cardiomyocyte Maintenance Medium (Stemcell Technologies, Canada) supplemented with 10% FBS and 5 µM ROCK inhibitor Y-27632, and plated at a 1:2 ratio on 24-well culture plates (SPL Life Science, Republic of Korea) precoated with Matrigel (Matrigel hES-qualified matrix, Corning, USA). For plating iCMs on glass, wells of eight-well culture slide flasks (SPL Life Science, Republic of Korea) were pretreated with 1 mg/mL polyethyleneimine (PEI, branched, Sigma-Aldrich, USA) for 1 h at room temperature, rinsed three times with distilled water, and coated with Matrigel. iCMs were cultured in STEMdiff Cardiomyocyte Maintenance Medium according to the manufacturer’s instructions.

### 4.6. Maturation of iCMs

Maturation of metabolically selected iCMs was carried out via a short protocol over 4 days (days 14–18 of the experiment) or a long protocol over 22 days (days 18–40 of the experiment) by culturing under standard conditions (37 °C, 5% CO_2_) in media containing maturation-promoting supplements as follows.

*Short protocol:*

1. Medium 1—STEMdiff Cardiomyocyte Maintenance Medium (Stemcell Technologies, Canada), 100 nM T3 (Sigma-Aldrich, Cat.#T6397, USA), 1 µM Dexamethasone (injectable solution, KRKA, Novo Mesto, Slovenia), 100 ng/mL rhIGF-1 (Stemcell Technologies, Cat.#78022, Canada), 1000× Chemically Defined Lipid Concentrate (Gibco, Cat.#11905-031, USA).
2. Medium 2—STEMdiff Cardiomyocyte Maintenance Medium (Stemcell Technologies, Canada), 25 µM Etoposide (Selleckchem, Houston, TX, Cat.#S1225, USA), 1000× Chemically Defined Lipid Concentrate (Gibco, Cat.#11905-031, USA).

*Long protocol:*

1. Medium 3—RPMI-1640 with 11 mM Glucose (Gibco, Cat.#21870-076, USA), 100 nM Triiodothyronine (Sigma-Aldrich, Cat.#T6397, USA), 1 µM Dexamethasone (injectable solution, KRKA, Slovenia), 100 ng/mL rgIGF-1 (Stemcell Technologies, Cat.#78022, Canada), and 50× NeuroMax supplement (PanEco, Cat.#FR-0305, Russia)
2. Medium 4—Glucose-free DMEM (Gibco, Cat.#A14430-01, USA) supplemented with 3 mM D-Glucose (injectable solution, Armavir Biological Factory, Armavir, Russia), 4 mM L-Glutamine (Corning, Cat.#25-005-CI, USA), 100× Non-essential amino acids for MEM (PanEco, Cat.#F115/100p, Russia), 10 mM Sodium lactate (PanReac, Cat.#143397.1211, Spain), 5 µg/mL Vitamin B12 (injectable solution, Dalhimfarm, Khabarovsk, Russia), 0.82 µM Biotin (PanReac, Cat.#A0969.0001, Spain), 5 mM Creatine monohydrate (dietary supplement, Evalar, Biysk, Russia), 2 mM L-Taurine (intravitreal solution, Dalhimfarm, Russia), 2 mM L-Carnitine (injectable solution, Ellara, Pokrov, Russia), 0.5 mM Ascorbic acid (Sigma-Aldrich, Cat.#A4544-256, USA), 0.5 mg/mL rhAlbumin (eEnzyme, Cat.#HSA-1g, USA), 1000× Chemically defined lipid concentrate (Gibco, Cat.#11905-031, USA), 50× NeuroMax supplement (PanEco, Cat.#FR-0305, Russia), and 1% KnockOut SR (Gibco, Cat.#10828-028, USA).

All media were supplemented with 50 U/mL, 50 μg/mL penicillin-streptomycin (PanEco, Russia). Stock solutions of maturation factors were prepared according to the manufacturers’ instructions. A stock solution of creatine monohydrate was prepared in distilled water and sterilized by filtration through a 0.22 µm filter. Medium changes were performed every 2 days (short protocol) or every 3 days (long protocol) with 0.5 mL or 1 mL of culture media per cm^2^ of cultureware, respectively.

The unmodified STEMdiff Cardiomyocyte Maintenance Medium (Stemcell Technologies, Canada) was used as a control condition for all experiments in accordance with the manufacturer’s protocol for long-term maintenance of iPSC-derived cardiomyocytes.

### 4.7. Preparation of iCMs Lysates

Cells were detached from the substrate using 0.25% trypsin on day 18 or day 40 from the start of differentiation, resuspended to a single-cell suspension, and counted using an improved Neubauer counting chamber C-Chip (INCYTO, Republic of Korea). The cell suspension was centrifuged for 4 min at 200× *g* (Eppendorf Centrifuge 5804R, Germany), the supernatant was completely removed, and the cell pellets were frozen at −20 °C in two technical replicates. After thawing, the pellets were resuspended in lysis buffer (10 mM KH_2_PO_4_, 150 mM NaCl, pH 7.4, 0.1% Triton X-100, phenylmethylsulfonyl fluoride, aprotinin) at a ratio of ≥5 × 10^3^ cells/µL buffer and processed by sonication using a Misonix X-400 Ultrasonic Liquid Processor (Misonix, Farmingdale, NY, USA). For further analysis by sandwich-type fluoroimmunoassay (FIA), cell lysates were diluted in antigen buffer (20 mM Tris-HCl, 150 mM KCl, 5 mM CaCl_2_, 7.5% bovine serum albumin (BSA), and pH 7.5, 0.15% NaN_3_). Samples containing ≥0.05 × 10^6^ cells were prepared in two dilutions: 1:50 and 1:200. Samples with <0.05 × 10^6^ cells were diluted once at a 1:4 ratio. Each dilution was analyzed in two technical replicates.

### 4.8. Sandwich Fluoroimmunoassay (FIA)

The concentrations of ssTnI, cTnI, and cTnT in lysates were measured by FIA using the antibody pairs skTnI58-skTnI27 (capture and detection antibodies, respectively) for ssTnI, Y306-Y603 for cTnI, and 329-406 for cTnT. Calibrators included ssIC for the skTnI58-skTnI27 system and cITC for the Y306-Y603 and 329-406 systems. The list of antibodies used is provided in Table 2. For analysis, monoclonal antibodies (mAbs) at 2 µg/mL were immobilized onto the surface of polystyrene 96-well plates (Greiner Bio-One, Kremsmünster, Austria) in buffer containing 10 mM KH_2_PO_4_, 150 mM NaCl, pH 7.4, in a volume of 50 µL per well. After incubation for 40 min at 25 °C with constant shaking, the wells were washed with wash buffer (10 mM Tris-HCl, pH 7.8, 0.9% NaCl, 0.025% Tween 40, 0.05% NaN_3_) using a PlateWasher instrument (Perkin Elmer, Springfield, IL, USA). Next, europium–chelate–conjugated mAbs were added in 25 µL volume in buffer containing 50 mM Tris-HCl, pH 7.8, 0.9% NaCl, 0.5% BSA, 0.01% Tween 40, 0.5% NaN_3_, followed by 25 µL of test samples diluted in antigen buffer (20 mM Tris-HCl, 150 mM KCl, 5 mM CaCl_2_, 7.5% BSA, pH 7.5, 0.15% NaN_3_). The mixture was incubated at 25 °C with constant shaking for 40 min and then washed again with wash buffer. Subsequently, 50 µL of enhancement solution (0.1 M CH_3_COOH, pH 3.2, 50 µM trioctylphosphine oxide, 50 µM 4,4,4-trifluoro-1-(2-naphthyl)-1,3-butanedione, 0.1% Triton X-100) was added to each well, incubated for 10 min at 25 °C with constant shaking, and fluorescence intensity was measured in the wells using a Victor 1420 Multilabel Counter (Perkin-Elmer, Waltham, MA, USA). To construct calibration curves, serial dilutions of ssIC or cITC were prepared in the concentration range of 0.08–80 ng/mL (troponin I concentration in complex).

### 4.9. Calculation of the cTnI/ssTnI Ratio in iCMs

The quantitative content of ssTnI, cTnI, and cTnT in cell lysates was determined using the concentration values (ng/mL) of ssTnI, cTnI, and cTnT in the tested samples. The amount of protein (ng) was normalized to one million cells (million cells) collected for lysis. The resulting values were used to calculate the cTnI/ssTnI ratio.

### 4.10. Immunocytochemical Staining (ICC)

Fixation of iCM cultures with approximately more than 50% confluence was performed on days 20, 22, and 40 from the start of differentiation: cells were washed once with Hank’s solution, incubated for 15 min in 4% paraformaldehyde solution (PanReac, Spain), washed with Hank’s solution, permeabilized for 10 min with 0.2% Triton X-100 solution (Sigma-Aldrich, USA), incubated for 30 min in blocking solution containing 0.1% Tween20 (PanReac, Spain) in PBS (PanEco, Russia) (PBS-T), 5% goat serum (Gibco, USA), and 5% fetal bovine serum (FBS) (Himedia, India), then stained overnight at 4 °C with primary monoclonal antibodies (see Table 2) diluted in blocking solution. Primary antibodies were washed three times for 5 min each with 0.1% PBS-T solution and then stained with secondary antibodies (see Table 2) diluted in 0.1% PBS-T for 30 min in the dark at room temperature. Secondary antibodies were washed twice for 5 min with 0.1% PBS-T, cells were incubated for 10 min in 100 ng/mL DAPI solution (Sigma-Aldrich, USA) in the dark at room temperature, washed once with PBS (PanEco, Russia), and mounted with PBS (PanEco, Russia) containing 0.02% NaN_3_ (Sigma-Aldrich, USA). Preparations on slides were mounted using Mowiol mounting medium (Sigma-Aldrich, USA). Microphotographs of cells were obtained using an inverted fluorescence microscope Olympus IX53F (Olympus, Tokyo, Japan) with cellSens Standard Version 1.11 software (Olympus, Japan), and a direct fluorescence microscope Nikon Eclipse Ni (Nikon, Tokyo, Japan) with NIS-Elements BR Version 5.30.06 software (Nikon, Japan) at the same excitation light intensity and exposure settings. Image processing was performed using open-source ImageJ2 Version 2.9.0 and GIMP Version2.10 software.

### 4.11. Flow Cytometry (FC)

On day 22 from the start of differentiation, iCMs were detached from the substrate using 0.25% trypsin. The resulting suspension was filtered through a 100 µm pore-size cell strainer (Nest, Shenzhen, China), and 0.25 × 10^6^ cells were taken for staining. Sample preparation was conducted at room temperature with cell pelleting by centrifugation using a bucket rotor (Eppendorf Centrifuge 5804R, Germany) at 300 g for 3 min. Cells were fixed for 10 min with 2% paraformaldehyde (PanReac, Spain), washed once with Hank’s solution (PanEco, Russia), permeabilized for 10 min with 0.2% Triton X-100 (Sigma-Aldrich, USA), incubated for 30 min in blocking solution containing 0.1% Tween20 (PanReac, Spain) in PBS (PanEco, Russia) (PBS-T), 5% goat serum (Gibco, USA), and 5% fetal bovine serum (FBS) (Himedia, India), then primary monoclonal antibodies against cTnT (see Table 2) were added and incubated for 1 h at room temperature. Primary antibodies were washed twice for 5 min with 0.1% PBS-T, and stained with secondary antibodies (see Table 2) diluted in 0.1% PBS-T for 30 min in the dark at room temperature. Secondary antibodies were washed twice for 5 min in PBS (PanEco, Russia), and the pellet was resuspended in 100 µL PBS (PanEco, Russia). Sample analysis was performed on a NovoCyte 3000 Flow Cytometer (ACEA Biosciences, San Diego, CA, USA) using ACEA NovoExpress Version 1.5.0 software (ACEA Biosciences, USA). The absence of nonspecific binding of secondary antibody was preliminarily confirmed on iCMs (Supplementary Figure S1). cTnT^+^-iCMs were gated on density plots by fluorescence intensity of the used marker (Alexa Fluor 488, Molecular Probes Inc., Eugene, OR, USA) and forward scatter (FSC) relative to autofluorescence of unstained iCMs.

### 4.12. Statistical Data Analysis

Data are presented as mean ± standard deviation (SD). Statistical analysis was performed using GraphPad Prism software (version 8.4.0). Significance for pairwise comparisons was calculated using ordinary one-way ANOVA with Dunnett’s post hoc test for normally distributed samples. A *p*-value < 0.05 was considered statistically significant.

## 5. Conclusions

We established and characterized a new human iPSC line, IPSAVE2S, demonstrated its high efficiency in differentiating into ventricular iCMs, and tested four distinct 2D culture media aimed at enhancing cardiomyocyte maturation. Our measurements of ssTnI and cTnI content are consistent with previous reports indicating that, in the absence of external stimuli, iCMs retain a fetal-like phenotype.

We show for the first time that the multicomponent metabolic maturation medium MM-1—formulated with reduced glucose, elevated lactate, essential amino acids, vitamin B12, biotin, creatine, taurine, carnitine, ascorbic acid, serum replacement, recombinant human albumin, defined fatty acids, and NeuroMax—promotes cTnI expression at the protein level and facilitates structural maturation in localized iCMs clusters. MM-1–based maturation may be readily integrated into routine protocols for generating human iPSC-derived cardiac models suitable for single-cell applications such as electron microscopy, confocal imaging, or patch-clamp electrophysiology. In addition to biochemical stimulation, the future integration of various in vitro maturation strategies—including iCMs electrostimulation and heart-on-a-chip technology—could significantly accelerate the elucidation of the fundamental principles governing cardiomyocyte maturation.

Among future strategies, targeting gene regulatory pathways that drive the transition to the postnatal cardiomyocyte state appears particularly promising. However, the identification of key in vivo maturation triggers remains a significant and interdisciplinary challenge in developmental cardiobiology.

## Figures and Tables

**Table 1:** Culture media and supplements used for human iCMs maturation.

Name	Basal Medium	Maturation Factors	Maturation Protocol
Medium 1	STEMdiff Cardiomyocyte Maintenance Medium	Triiodothyronine (T3), dexamethasone, insulin-like growth factor 1 (IGF-1), chemically defined lipid concentrate	Short (4-day)
Medium 2	STEMdiff Cardiomyocyte Maintenance Medium	Etoposide, chemically defined lipid concentrate	Short (4-day)
Medium 3	RPMI-1640 with Glucose	T3, dexamethasone, IGF-1, NeuroMax supplement (NeuroMax)	Long (22-day)
Medium 4 (MM-1)	DMEM without Glucose	D-glucose, L-glutamine, non-essential amino acids, sodium lactate, vitamin B12, biotin, creatine monohydrate, L-taurine, L-carnitine, ascorbic acid, albumin, chemically defined lipid concentrate, NeuroMax, KnockOut serum replacement (KnockOut SR)	Long (22-day)

**Table 2:** Antibodies used for FIA, ICC staining, and flow cytometry of iCMs.

Target Protein	Antibody	Manufacturer, Cat.#, or Reference	Dilution
FIA			
ssTnI	skTnI58	[55]	-
	skTnI27	[55]	-
cTnI	Y306	HyTest #RC4T21	-
	Y603	HyTest #RC4T21	-
cTnT	329	HyTest #4T19cc	-
	406	HyTest #4T19cc	-
ICC			
ssTnI	skTnI30	[55]	1:1000
cTnI	MF4	HyTest #4T21	1:1000
Goat anti-Mouse IgG (H + L)	Alexa Fluor 555-conjugated secondary antibody	Invitrogen #A-21422	1:1000
FC			
cTnT	406	HyTest #4T19CC	1:1000
Goat anti-Mouse IgG (H + L)	Alexa Fluor 488-conjugated secondary antibody	Invitrogen #A-11001	1:1000

## Data Availability

The original contributions presented in this study are included in the article/Supplementary Material. Further inquiries can be directed to the corresponding author.

## Supplementary Materials

The following supporting information can be downloaded at: https://www.mdpi.com/article/10.3390/ijms26157248/s1.

## Abbreviations

The following abbreviations are used in this manuscript:

ATP	adenosine triphosphate
BSA	bovine serum albumin
CDM3L	chemically defined Medium 3 without glucose with lactate
cITC	ternary human cardiac troponin complex (I-T-C)
cTnI	cardiac troponin I
cTnT	cardiac troponin T
DAPI	4′,6-diamidino-2-phenylindole
DMEM	Dulbecco’s modified Eagle medium
DMSO	dimethyl sulfoxide
DNA	deoxyribonucleic acid
EBs	embryoid bodies
EDTA	ethylenediaminetetraacetic acid
FBS	fetal bovine serum
FC	flow cytometry
FIA	fluoroimmunoassay
FSC	forward scatter
hc-MYC	human cellular myelocytomatosis oncogene
hKLF4	human Krüppel-like factor-4
hOCT3/4	human octamer-binding transcription tactor-3/4
hPSC	human pluripotent stem cells
hSOX2	human SRY-box transcription factor 2
ICC	immunocytochemical staining
iCMs	induced pluripotent stem cell-derived cardiomyocytes
IGF-1	insulin-like growth factor 1
iPSCs	induced pluripotent stem cells
MEM	minimum essential medium
MM-1	metabolic maturation medium 1
PBS	phosphate-buffered saline
PEI	polyethyleneimine
ROCK	rho-associated protein kinase
RPMI	Roswell Park Memorial Institute
SEM	standard error of the mean
skTnI	skeletal troponin I
SOX17	SRY-box transcription factor 17
SR	serum replacement
ssIC	binary human slow skeletal troponin complex (I-C)
ssTnI	slow skeletal troponin I
STR	short tandem repeat
T3	triiodothyronine
TNNI3	troponin I3, cardiac type
TRA-1-81	podocalyxin-like protein 1
