# Supplementary material for: Metabolic Culture Medium Enhances Maturation of Human iPSC-Derived Cardiomyocytes via Cardiac Troponin I Isoform Induction
Source: Int J Mol Sci. 2025 Jul 26;26(15):7248. doi: 10.3390/ijms26157248 (PMC12346743; doi:10.3390/ijms26157248)
Supplement: Supplementary file 1 [file ijms-26-07248-s001.zip › ijms-3767080-supplementary.pdf]

SUPPLEMENTARY MATERIAL

Table S1. RCPCMi013-A (IPSAVE2S) iPS cell line passport.

No.	Characteristic	Data
1	Unique Identifier	RCPCMi013-A
2	Alternative Name	IPSAVE2S
3	Institution	Lopukhin Federal Research and Clinical Center of Physical-Chemical Medicine, Moscow, Russia
4	Ethics Committee Approval	Study approved by the Ethics Committee of the Lopukhin Federal Research and Clinical Center of Physical-Chemical Medicine; protocol No. 1 dated 1 June 2021
5	Cell Type	iPSCs
6	Organism Species	Human
7	Donor Information	Age: 46<br>Sex: Male<br>Ethnicity: Caucasian
8	Original Cell Type	Skin fibroblasts
9	Biomaterial Collection Date	2022
10	Reprogramming Method	Sendai virus-based (non-integrative)
11	Reprogramming Factors	OCT3/4, SOX2, KLF4, C-MYC
12	Clonality	Clonal, clone 2S
13	Disease Status	None
14	Morphology	Colonies exhibiting typical human pluripotent stem cell morphology
15	Pluripotency Confirmation	Verified by embryoid body formation assays, spontaneous differentiation into derivatives of all three germ layers, and specific marker expression analysis
16	Karyotype	46, XY
17	Contamination Testing	Mycoplasma not detected
18	Application Area	Cell biology research

19	Culture Method	On Matrigel substrate (Matrigel hES-qualified matrix, Corning)
20	Culture Medium	GibriS-8 (PanEco) + mTeSR1 (Stemcell Technologies) mixed at 4:1 ratio, supplemented with 50 U/mL and 50 μg/mL penicillin-streptomycin
21	Temperature, °C	37
22	CO_2 Concentration, %	5
23	Passaging Method	Enzymatic, 0.05% Trypsin-EDTA (Gibco or PanEco)
24	Passaging Ratio	1:4 - 1:5
25	Cryopreservation	90% FBS (Himedia), 10% DMSO (PanEco), 5 μM ROCK inhibitor Y-27632 (Stemcell Technologies)
26	Storage Conditions	Liquid nitrogen
27	Registry Account	HPSReg ID: RCPCMi013-A
28	Date of Depositing	05.02.2025

Table S2. Data on cell count, troponins concentration, normalized troponins content and the resulting cTnI/ssTnI ratio in the analyzed iCMs samples.

No	Sample	10^6 cells	Lysate volume, μL	Protein concentration, ng/mL			Protein content in lysate, ng			Normalized protein content, ng/10^6 cells			
				ssTnI	cTnI	cTnT	ssTnI	cTnI	cTnT	ssTnI	cTnI	cTnT	cTnI/ ssTnI
Short-term maturation													
1	Control #1 [1]	1.249	200	210.2	6.6	3378.5	42.0	1.31	675.7	33.66	1.05	541.98	0.031
2	Control #2	1.734	200	177.5	7.5	3274.4	35.5	1.51	654.9	20.47	0.87	377.67	0.043
3	Medium 1 #1	1.500	200	225.3	10.6	3294.3	45.1	2.12	658.9	30.04	1.41	439.24	0.047
4	Medium 1 #2	1.275	200	250.1	12.8	4093.1	50.0	2.56	818.6	39.24	2.01	642.05	0.051
5	Medium 2 #1	1.125	200	277.9	16.4	4100.7	55.6	3.28	820.1	49.40	2.91	729.01	0.059
6	Medium 2 #2	1.125	200	281.8	16.2	4973.3	56.4	3.25	994.7	50.09	2.89	884.14	0.058
Long-term maturation													
1	Control #1	0.430	100	176.21	10.14	3329.58	17.62	1.014	332.96	40.98	2.36	774.32	0.058

2	Control #2	0.420	100	116.06	9.07	3170.41	11.61	0.907	317.04	27.63	2.16	754.86	0.078
3	Medium 3 #1	0.100	100	0.41	0.12	5.97	0.04	0.012	0.60	0.41	0.12	5.97	0.306
4	Medium 3 #2	0.100	100	1.12	0.11	12.93	0.11	0.011	1.29	1.12	0.11	12.93	0.100
5	Medium 4 #1	0.070	100	18.15	0.35	90.83	1.82	0.035	9.08	25.93	0.50	129.75	0.019
6	Medium 4 #2	0.025	100	8.67	0.42	29.27	0.87	0.042	2.93	34.70	1.69	117.09	0.049

[1] #1, #2 – technical replicates.

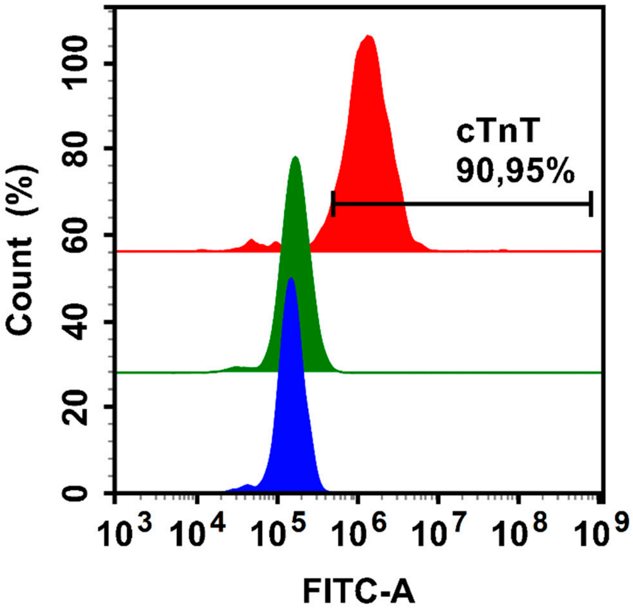

Figure S1. Flow cytometry data on fluorescence intensity of unstained (green), secondary antibody only stained (blue) and cTnT-stained (red) AVE2S-iCMs (passage 1, day 23). The histogram demonstrates the absence of secondary antibody nonspecific binding in human iCMs.
